# Vacuolar Sorting Receptor-Mediated Trafficking of Soluble Vacuolar Proteins in Plant Cells

**DOI:** 10.3390/plants3030392

**Published:** 2014-08-25

**Authors:** Hyangju Kang, Inhwan Hwang

**Affiliations:** 1 Department of Life Sciences, Pohang University of Science and Technology, Pohang 790-784, Korea; 2 Division of Integrative Biosciences and Biotechnology, Pohang University of Science and Technology, Pohang 790-784, Korea

**Keywords:** vacuolar sorting receptors, protein trafficking to vacuoles, sorting signals, molecular machinery, soluble vacuolar proteins

## Abstract

Vacuoles are one of the most prominent organelles in plant cells, and they play various important roles, such as degradation of waste materials, storage of ions and metabolites, and maintaining turgor. During the past two decades, numerous advances have been made in understanding how proteins are specifically delivered to the vacuole. One of the most crucial steps in this process is specific sorting of soluble vacuolar proteins. Vacuolar sorting receptors (VSRs), which are type I membrane proteins, are involved in the sorting and packaging of soluble vacuolar proteins into transport vesicles with the help of various accessory proteins. To date, large amounts of data have led to the development of two different models describing VSR-mediated vacuolar trafficking that are radically different in multiple ways, particularly regarding the location of cargo binding to, and release from, the VSR and the types of carriers utilized. In this review, we summarize current literature aimed at elucidating VSR-mediated vacuolar trafficking and compare the two models with respect to the sorting signals of vacuolar proteins, as well as the molecular machinery involved in VSR-mediated vacuolar trafficking and its action mechanisms.

## 1. Introduction

Plant cells contain a variety of endomembrane compartments. Of these, the vacuole is the most prominent organelle, occupying up to 90% of the cellular volume in mesophyll cells. Two different types of vacuoles exist in plant cells: the lytic vacuole in vegetative cells and the protein storage vacuole (PSV) in seed cells [1,2]. These vacuoles play many important roles, such as the degradation of waste materials, ion and metabolite storage, and the maintenance of turgor pressure. To perform these activities, a large number of proteins localized to both the lumen and tonoplast are required. In plant cells, nascent vacuolar proteins are initially targeted to the endoplasmic reticulum (ER) and are subsequently transported to the vacuole via multiple routes depending on the individual proteins.

Of these multiple routes, the most prominent employs multiple intermediate organelles, *i.e.*, the Golgi apparatus, trans-Golgi network (TGN), and prevacuolar compartment (PVC) [3,4,5,6,7,8,9]. In this route, the process of protein trafficking from the ER to the vacuole comprises many distinct steps, each of which requires a large number of protein factors. In fact, numerous molecular factors involved in this route have been characterized at the molecular and cellular levels. One of these factors is the vacuolar sorting receptor (VSR), which plays a crucial role as a sorting receptor for soluble vacuolar proteins during their trafficking to the vacuole. VSRs are highly conserved among plant species, including BP80 (binding protein 80 kD) in pea, PV72 in pumpkin, and seven VSRs in Arabidopsis [8,10,11,12,13,14]. VSRs are type I membrane proteins, which consist of a large N-terminal luminal domain, a transmembrane domain (TMD), and a short C-terminal cytosolic tail [10,15]. The luminal domain binds to soluble vacuolar proteins, while the cytosolic tail interacts with various trafficking components for vesicle formation and for its own recycling [8,16,17,18,19,20].

Alternatively, certain vacuolar proteins are transported directly from the ER to the vacuole via a Golgi-independent route [21,22,23]. In particular, multiple tonoplast proteins including α-tonoplast intrinsic protein, calcineurin B-like protein 6, the two-pore potassium channel TPKb, the vacuolar H^+^-ATPase VHA-a3, and the H+-pyrophosphatase AVP1 are transported via a Golgi-independent route [21,22,23,24,25]. In pumpkin seed cells, precursor-accumulating (PAC) vesicle-mediated trafficking transports proteins from the ER to the PSV via a Golgi-independent route [26]. Tonoplast proteins can also be transported by another route; the sucrose transporter SUC4 is transported from the ER to the TGN through the Golgi apparatus and is then directly targeted from the TGN to the tonoplast without passing through the PVC in an adaptor protein complex (AP)-3-dependent manner [27].

In this review, we focus on the VSR-mediated trafficking of soluble proteins to the vacuole. For other routes of vacuolar trafficking, we recommend other recent reviews [28,29,30,31]. Here, we summarize recent advances in elucidating VSR-mediated trafficking of soluble proteins to two types of vacuoles in plants, *i.e.*, lytic vacuoles and PSVs, and we discuss the two models describing this process.

## 2. Involvement of VSRs in Trafficking of Soluble Vacuolar Proteins to Lytic Vacuoles and PSVs

VSRs were originally identified as abundant proteins in the CCV fraction purified from plant extract [10]. Subsequently, many VSR homologs have been identified in association with the vacuolar trafficking process in various plant species, and they were thus proposed to function as sorting receptors of soluble vacuolar proteins [13,32,33]. The biological role of VSRs has been tested by examining protein trafficking to the vacuole. The protoplast system has been widely used to investigate the role of VSRs in lytic vacuolar trafficking. Transient expression of dominant negative mutant forms of AtVSR1 and AtVSR2 causes secretion and/or inhibition of various coexpressed vacuole-destined proteins, such as Spo:GFP (a fusion protein consisting of a sorting signal of sporamin protein from sweet potato (*Ipomoea batatas*) and green fluorescent protein (GFP)), AALP:GFP (a fusion of Arabidopsis aleurain-like protein and GFP), and Spo:amylase (a fusion of sporamin and amylase, destined for the lytic vacuole) [18,19,34,35]. These studies have provided strong evidence that VSRs are sorting receptors of soluble lytic vacuolar proteins, which was confirmed by genetic studies; in *atvsr1atvsr4* double-mutant plants, a small amount of AALP is secreted into the apoplast in leaf tissues [13]. In addition, in various *atvsr* single and double mutants (*atvsr1*, *arvsr1atvsr3* and *atvsr1atvsr4*), vacuolar trafficking capacity is reduced to varying degrees depending on the genotype, although these mutants do not show any noticeable defects in vacuolar trafficking in intact leaf tissues [35]. The defect in lytic vacuolar trafficking in *atvsr1atvsr4* double-mutant plants was complemented by transient expression of AtVSR1 or AtVSR4, further confirming that these proteins are involved in lytic vacuolar trafficking. Their role in lytic vacuolar protein sorting has also been directly supported by numerous ways; ER-retained soluble PV72 (PV72-HDEL) caused accumulation of AtALEU in the ER in Arabidopsis transgenic plants [36]. Amy-spo, a chimeric vacuolar cargo consisting of the N-terminal region of sporamin and amylase, was secreted into the apoplast when a mutant form of BP80, full-length BP80-Y612A, which was mistargeted to the plasma membrane was coexpressed in tobacco protoplasts [18]. In cultured cell lines of Arabidopsis, expression of the luminal domain of AtVSR1 caused co-secretion of various vacuolar proteins into the medium [37].

In addition, the role of VSRs in PSV trafficking has been confirmed by genetic studies. In a knock-out mutant of *AtVSR1*, significant amounts of 12S globulins and 2S albumins accumulate as precursors and are partially secreted into the extracellular matrix [33]. Zouhar *et al.* [13] subsequently confirmed this observation and demonstrated that of the seven AtVSR isoforms, two isoforms, AtVSR3 and AtVSR4, are also involved in PSV trafficking and are functionally redundant to AtVSR1. Interestingly, these *atvsr* mutants have smaller PSVs than the wild type, which may be due to the reduced levels of PSV proteins in the PSVs resulting from defects in PSV trafficking. However, single and double mutants of *atvrs1*, *atvsr3*, and *atvsr4* do not show any obvious defective phenotype in their vegetative tissues.

While the physiological roles of these AtVSR isoforms have been elucidated, the roles of AtVSR5 and AtVSR6 are not yet known. These two proteins also localize primarily to the PVC in protoplasts, as does AtVSR1 [35,38]. However, in contrast to other *atvsr* mutants, *atvsr5atvsr6* double-mutant plants did not exhibit defective trafficking of protein to the two vacuoles (lytic vacuole and PSV) when two lytic vacuolar cargoes, sporamin:GFP and AALP:GFP, and two PSV proteins, 12S globulins and 2S albumins, were examined [13,35]. The difference between these two VSR isoforms (VSR5 and VSR6) and other VSR isoforms in terms of protein trafficking to the vacuoles stems from the difference in their luminal domains; when the luminal domains of AtVSR1 and AtVSR5 were swapped, the vacuolar trafficking activity of the resulting mutants was determined by the luminal domain [35]. These results indicate that the luminal domain is involved in the specificity determination of AtVSR isoforms. However, we cannot exclude the possibility that AtVSR5 and AtVSR6 may also play a role in sorting vacuolar cargoes other than those examined. Further studies are necessary to determine the exact role of these two isoforms in plant cells.

## 3. Vacuolar Sorting Signals and Their Interactions with VSRs

When VSRs function as sorting receptors, one of their most important activities is the specific recognition of vacuolar proteins among the numerous organellar proteins that are simultaneously transported through the endomembrane compartments. Vacuolar proteins contain a specific sequence motif, the sorting signal, which is required for specific recognition by VSRs. The sorting signals of various vacuolar proteins are classified into two groups, sequence-specific vacuolar sorting signal (ssVSS) and C-terminal vacuolar sorting signal (ctVSS). The ssVSSs show a consensus sequence while the ctVSSs are poorly defined but generally composed of hydrophobic amino acids [39]. ssVSSs, which have been identified from lytic vacuolar proteins, such as barley proaleurain and sweet potato sporamin, include NPIR or similar sequences [6,40,41,42]. Indeed, peptides containing ssVSSs specifically bind to VSRs *in vitro* [10,16]. Consistent with the role of VSRs in the sorting of PSV proteins, the ctVSSs of Brazil nut 2S albumin and Arabidopsis 12S globulin strongly bind to BP80 and AtVSR1, respectively, while the C-terminal sorting signal of barley lectin shows weak binding [10,16,33]. These observations raise the intriguing question of how VSRs bind to two different types of vacuolar sorting signals, the ssVSS and ctVSS. One possibility is that they are recognized by different cargo binding sites in the luminal domain.

The mechanism underlying the specific recognition of sorting signals by VSRs has been elucidated at the molecular level. VSRs must bind to cargoes at the donor compartment and release them at the acceptor compartment. Ca^2+^ ion plays a crucial role in cargo binding to VSRs *in vitro*; cargo binding to VSRs is enhanced by high concentrations of Ca^2+^ [10,43,44,45]. Consistent with this finding, VSRs contain a Ca^2+^-binding motif, the epidermal growth factor (EGF)-like motif, at the luminal domain. Moreover, Ca^2+^-binding to the EGF-like motif is crucial for its interaction with other proteins [46]. Indeed, a luminal domain lacking the EGF-like motif exhibited a low level of cargo binding. These results support the notion that Ca^2+^ is involved in the interaction between cargoes and the luminal domains of VSRs [44,47]. However, it is still not fully understood how Ca^2+^ binding to the EGF-like motif contributes to cargo binding to the luminal domain.

Another important factor for the interaction between cargoes and their receptors is pH. In animal cells, the lysosomal cargo receptor mannose-6-phosphate (M6P) receptor (MPR) binds to M6P (the sorting signal of lysosomal cargoes) at the TGN and releases these cargoes at the late endosome [48]. In this process, the pH of these compartments is crucial for their interaction and dissociation; the pH of the TGN and the late endosomes is 6.0 and 5.5, respectively [49]. Therefore, the organelle in which cargo binding takes place has a higher pH than that for cargo release, indicating that a higher pH is favorable for cargo binding and a lower pH is favorable for cargo release. For VSRs, *in vitro* experiments have shown that the optimal pH for cargo binding to VSRs is pH 6–7, while they dissociate at pH 4 [10,47]. Currently, the pH levels of the plant organelles are not yet clearly defined; in fact, the results of two recent studies examining the pH of endomembrane compartments differ [50,51]. However, neither of these studies supports the *in vitro* result showing that cargo release from VSRs occurs at pH 4.0; thus other conditions, including the Ca^2+^ concentrations in the lumens of the compartments, may contribute to cargo binding to and release from VSRs during vacuolar trafficking.

## 4. Molecular Mechanisms of VSR-Mediated Vacuolar Trafficking

Extensive studies have been carried out investigating the molecular mechanisms of VSR-mediated vacuolar trafficking in various plant species. These studies have resulted in the proposal of two different models describing VSR-mediated vacuolar trafficking: VSR-mediated trafficking from the TGN to the PVC (Model I) and VSR-mediated trafficking from the ER to the TGN (Model II). These models are significantly different, particularly with respect to the locations of cargo binding to and release from the VSRs, as well as the use of carrier vesicles.

### 4.1. Model I: VSR-Mediated Trafficking from the TGN to the PVC

**Figure 1:**
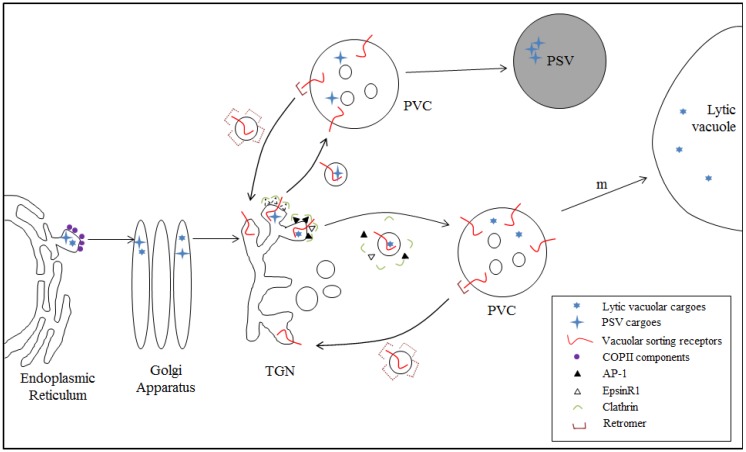
Vacuolar sorting receptor (VSR)-mediated soluble cargo transport from the *trans*-Golgi network (TGN) to the prevacuolar compartment (PVC): Model I. Nascent soluble vacuolar proteins are initially targeted to the endoplasmic reticulum (ER). Subsequently, they are transported to the Golgi apparatus in a COPII-dependent manner. Traveling from *cis*-Golgi to the TGN occurs via cisternal maturation. At the TGN, VSRs recognize their cargoes and receptor-cargo complexes are packaged into clathrin-coated vesicles with the help of AP-1 and/or EpsinR1. Clathrins and adaptor proteins dissociate from the clathrin-coated vesicles (CCVs) after vesicle release. After fusion of vesicles harboring vacuolar cargoes with the PVC, VSRs release their cargoes. VSRs are recognized by VPS29-containing retromers and recycle back to the TGN for the next round of cargo sorting. The exact mechanism of how retromers recognizes VSRs at the PVC remains elusive. It is also unknown whether retromers dissociate from the recycling vesicles in plants; thus retromers on the recycling vesicle are indicated with dotted lines. VSRs are also involved in trafficking of proteins to the PSV at the TGN via CCVs. However, it is not known which adaptors are involved in this pathway. It is also not clear whether the PVCs for the lytic vacuole and PSV are the same compartment or two different organelles. m, process occurring through maturation.

Model I closely resembles the models describing lysosomal and vacuolar trafficking in animal cells and yeast, respectively, whereas Model II appears to be specific to plant cells. One of the key differences in the two models is the localization of VSRs. In Model I, VSRs primarily localize to the PVC, while a significant proportion localize to the TGN. Indeed, immunogold labeling, immunostaining using anti-VSR antibody, and live cell imaging of GFP- or RFP-tagged VSRs have revealed that VSRs localize primarily to the PVC, with a significant proportion localizing to the TGN or trans-face of the Golgi [52,53,54,55]. The TMD and cytosolic tail of VSRs are sufficient for their localization to the PVC, because GFP-VSRs (with the luminal domain replaced with GFP) localize to the PVC [34,38,54]; thus, in Model I, VSRs bind to vacuolar cargoes at the TGN and release them at the PVC, thereby cycling between the TGN and PVC (Figure 1). In this model, the behavior of VSRs is conceptually similar to that of the sorting receptors MPRs in animal cells and Vps10p in yeast [56,57,58,59]. Indeed, the recycling of VSRs from the PVC to the TGN has been confirmed; The significant amounts of Spo:GFP and AALP:GFP were accumulated to the TGN in *vps29* mutant. Moreover, in the *vps29* mutant plants, VSRs accumulate to the PVC even when anterograde trafficking is inhibited by LatB, an inhibitor of actin filaments [60].

A large number of proteins function either directly or indirectly in VSR-mediated vacuolar trafficking. These protein factors include AtVTI11, EPSIN1 (renamed EpsinR1), AP-1, clathrin, actin filaments, and VPS29. Their physiological roles in VSR-mediated vacuolar trafficking have been confirmed genetically; like the *vsr1vsr4* double mutant, *atvti11*, *vps29*, *epsinr1*, and *ap1m2* mutant plants exhibit a defect in protein trafficking to the PSV and/or lytic vacuole. Of these proteins, EpsinR1 and AP-1 are monomeric and heterotetrameric adaptors of CCVs, respectively, thus raising the possibility that CCVs function as carriers during VSR-mediated vacuolar trafficking. Indeed, AtVSR1 interacts with EpsinR1. AP-1 may also have a direct interaction with VSRs since VSRs have an AP-1 binding motif, the YXXΦ motif, at their cytosolic tail. In fact, these proteins together with clathrin appear to form an interaction network at the TGN that is crucial for CCV formation [17,52,61]. In addition, μ-adaptin of AP-4 interacts with the cytosolic tail of VSR2 [20]. Another important interaction is the homomeric interaction between VSRs via a motif in the cytosolic tail [19], which is similar to oligomerization of MPRs in animal cells [62]. Therefore, overall, VSR-mediated vacuolar trafficking is conceptually similar to CCV-mediated trafficking of lysosomal cargoes in animal cells. In the interaction of VSRs with these proteins, the YXXΦ motif in the cytosolic tail is crucial; substitution of the Y residue of the YMPL motif with alanine causes mislocalization to the plasma membrane in addition to accumulation at the TGN [8,18,63,64]. This observation again supports the idea that VSRs are loaded into CCVs via the YXXΦ binding factor AP-1.

VSRs require additional molecules for their action. The majority of VSRs localize to the PVC. These VSRs must recycle to the TGN for cargo binding. In animal cells and yeast, retromer (a pentameric complex composed of two sorting nexins, VPS26, VPS29, and VPS35) is involved in the recycling of MPRs and Vps10p from the late endosome/PVC to the TGN [58,65,66,67]. All of the retromer components have been identified in plant cells. Moreover, VPS35, the receptor-binding subunit of retromer in animal cells and yeast [68], coimmunoprecipitates with Arabidopsis VSRs, and the VPS35/29/26 complex (cargo-selective subcomplex) localizes to the PVC [69]. Immunostaining experiments have suggested that SNXs and VPS29 localize to the PVC as well as the TGN [70,71,72,73]. Recently, however, both immunogold labeling and immunostaining experiments have demonstrated that SNX1 and SNX2a, as well as VPS29, localize to the TGN and not the PVC [74,75]. Currently, it is not easy to explain this inconsistency. Biologically active SNX1-XFP and VPS29-GFP are sensitive to both brefeldin A and wortmannin [70,71]. In addition, VPS29-GFP showed membrane localization in *snx1-1* and *snx2a-2snx2b-1* mutants. These results raise the possibility that retromer components localize to both the TGN and PVC, and they may not always function together [73]. Indeed, in mammalian cells, retromer localizes to both donor and acceptor membranes [76].

In addition, *vps29* mutant plants show defects in both PSV and lytic vacuolar trafficking [60,77]. A recent study has shown that VPS29 is crucial for recycling from the PVC to the TGN [60]. These results raise the possibility that retromer is involved in the recycling of VSRs from the PVC to the TGN. If this recycling indeed occurs, VSRs should have a specific sequence motif at the C-terminal cytosolic tail that can be recognized by retromer. Indeed, GFP-VSR2 (L615A), in which alanine is substituted for leucine in the YXXΦ motif, trafficks to the vacuole [8,18], raising the possibility that the YXXΦ motif also plays a role in retrograde trafficking. Similarly, yeast Vps10p, which also functions as a vacuolar sorting receptor, contains the YXXΦ motif, which is involved in recycling from the PVC to the TGN [78].

According to Model I, VSRs should bind to their cargoes at the TGN. Indeed, a large number of proteins involved in VSR-mediated trafficking localize to the TGN, including AtVTI11, EpsinR1, AP-1, and clathrin [17,79]. In addition, in protoplasts of *mag1-1/vps29* mutant plants, which have a defect in retrograde trafficking of VSRs from the PVC to the TGN, vacuolar cargoes accumulate to the TGN or are secreted into the medium [60]. However, a few critical questions should be answered before this model is conclusively accepted. As mentioned above, Ca^2+^ is an important cofactor for cargo binding to VSRs [10,45,47]. When the Ca^2+^ concentration was reduced from 1 mM to 10 μM, binding of the luminal domains of BP80 and AtVSR4 to a peptide containing the aleurain sorting signal dropped to 20% and 50%, respectively [45], indicating the importance of Ca^2+^ concentration for cargo binding to VSRs. The ER and Golgi contain 0.05–0.5 mM and 0.7–3 μM Ca^2+^, respectively [80,81]. If the Ca^2+^ concentration of the TGN is similar to that of its neighboring organelle, the Golgi, it is too low to enable a high level of binding of cargoes to VSRs. However, the exact concentration of Ca^2+^ at the TGN is unknown. In addition, pH also plays a critical role in the binding of cargoes to VSRs. Currently, the exact pH levels of the TGN and PVC in plants are not well established. Two recent studies reported conflicting data on the pH of these organelles. One study reported that the pH of the TGN and PVC are 6.3 and 6.2, respectively [51], whereas the other study reported that the pH of the TGN and PVC are 6.1 and 7, respectively [50]. The underlying reason for the differences observed in the pH levels of these organelles is not fully understood. In fact, both studies employed a similar pH sensor, ratiometric pHluorin. It is likely that a combination of two factors, pH and Ca^2+^ concentration, determines the binding of cargoes to VSRs. In addition, the homomeric interaction of VSRs may also contribute to the binding of cargoes because the effective concentration of the luminal domain involved in cargo binding is increased by this homomeric interaction [19].

Another important question is how cargoes are released from VSRs at the PVC in Model I. In fact, there is no direct evidence for vacuolar cargo dissociation from VSRs in the PVC. A peptide containing an N-terminal sorting motif binds to VSRs optimally at pH 6–7 and dissociates at pH 4. However, according to two recent studies showing that the pH level of the PVC is 6.2 or 7 [50,51], cargoes may not be easily released from VSRs at the PVC. It is likely that the dissociation of cargoes from VSRs may also be determined by a combination of two factors, pH and Ca^2+^ concentration. However, additional studies are necessary to confirm the dissociation of cargoes from VSRs in the PVC.

### 4.2. VSR-Mediated Transport from the ER to the TGN: Model II

**Figure 2:**
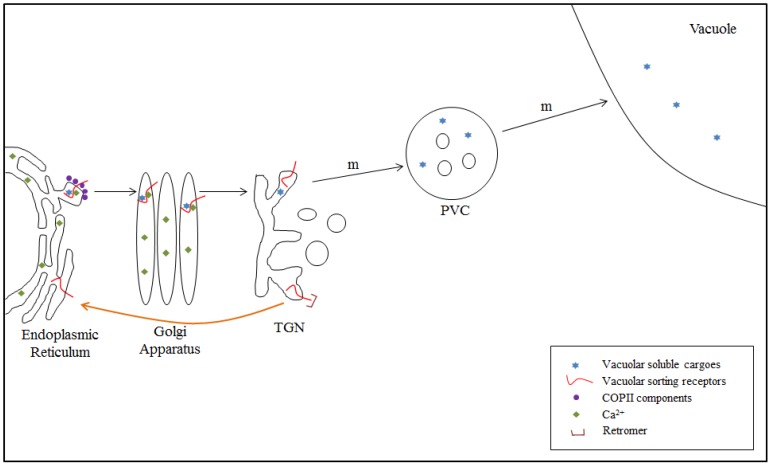
Vacuolar sorting receptor (VSR)-mediated cargo trafficking from the endoplasmic reticulum (ER) to the *trans*-Golgi network (TGN): Model II. After targeting of nascent vacuolar proteins to the ER, the folded vacuolar proteins bind to VSRs in the ER. Ca^2+^ plays a critical role in this binding. VSR-cargo complexes are transported to the cis-Golgi via COPII vesicles. The complexes are maintained until they reach the TGN, where the cargoes are released from the VSR due to the low concentrations of Ca^2+^. VSRs are selectively recycled back to the ER by retromer. Vacuolar cargo-enriched domains of the TGN mature into the prevacuolar compartment (PVC). The endosomal sorting complexes required for transport (ESCRT) machinery might be involved in this maturation step. m, process occurring through maturation.

Recently, a new model has been proposed for VSR-mediated vacuolar trafficking in plant cells. This model is based on several observations that cannot be explained by Model I. Niemes *et al.* [82] showed that coexpression of mutant forms of SNXs or RNAi knockdown of SNXs cause accumulation of GFP-BP80 (a chimeric protein in which the luminal domain of BP80 is replaced by GFP) at the ER together with soluble vacuolar cargoes, while the secretion of α-amylase and the targeting of the Golgi protein Man1-RFP are not affected. In addition, ER-retained VSR mutants and ER-localized soluble PV72 accumulate soluble vacuolar proteins, but not Golgi proteins, in the ER [36,82]; thus, VSRs may bind to their cargoes at the ER/Golgi but not at the TGN. High concentrations of free Ca^2+^ in the ER could be favorable for binding of VSRs to cargo proteins in the ER [80,81]. In another study, Niemes *et al.* [74] found that SNX1, SNX2a, and VPS29 of retromer (involved in the recycling of VSRs) localize to the TGN in Arabidopsis protoplasts and in root cells of Arabidopsis and tobacco, as revealed by immunogold labeling and immunostaining. Stierhof *et al.* [75] also provided evidence for the TGN localization of SNX1 and SNX2a in Arabidopsis root cells by immunogold labeling. In addition, transient expression of SNX1 or SNX2a mutants in protoplasts, as well as RNAi knockdown of SNXs caused mislocalization of BP80 to the TGN. These results support Model II, in which VSRs cycle between the ER and TGN (Figure 2).

Another important difference between the two models is the trafficking of vacuolar proteins from the TGN to the PVC. Model II proposes that the trafficking of vacuolar proteins from the TGN to the PVC occurs in a receptor-independent manner without any carriers. It has been proposed that the TGN is converted to the PVC via organelle maturation [74]. Indeed, maturation of the TGN to the PVC has been suggested by electron microscopy [83]. Subsequently, the PVC may fuse with the vacuole. Rab5-to-Rab7 replacement is crucial for the PVC-vacuole fusion in Arabidopsis. The MON1/SAND-CCZ1 complex was shown to be involved in this Rab replacement process [84,85,86].

Model II raises the question of how vacuolar proteins are sorted from secreted cargo proteins at the TGN if the cargoes are dissociated from VSRs at the TGN and the vacuolar cargoes are transported from the TGN to the PVC via maturation of the TGN into the PVC. In animal cells, it has recently been demonstrated that secretory proteins are actively sorted at the TGN in a Ca^2+^-dependent manner [87]. This active sorting involves Ca^2+^-ATPase SPCA1 and Ca^2+^-binding protein Cab45 at the membrane and in the lumen of the TGN, respectively [88,89,90,91]. In Arabidopsis, ECA3 (endoplasmic reticulum-type calcium ATPase 3) localizes to the Golgi/TGN/endosomes, and *eca3* mutant plants have a defect in transporting apoplastic peroxidases [92,93]. In addition, secretory vesicles form at the trans-side of the Golgi [94], which serves as a mechanism for discriminating secretory cargoes from vacuolar cargoes at the TGN. Moreover, sorting of secretory cargoes via a complex consisting of Cab45, Ca^2+^, and Ca^2+^-ATPase at the trans-Golgi may lead to a reduction in Ca^2+^ concentration at the TGN, thereby resulting in the release of vacuolar cargoes from VSRs. Based on these possibilities, Robinson and Pimpl [95] suggested that Ca^2+^-based vacuolar cargo sorting occurs at the TGN; thus, according to this model, soluble vacuolar proteins interact with VSRs at the ER and the cargo-VSR complexes are transported to the cis-Golgi via COPII vesicles together with secretory cargo proteins. At the trans-Golgi, secretory cargoes bind to the Ca^2+^-binding protein together with Ca^2+^-ATPase, while vacuolar cargoes remain in association with VSRs. Secretory cargoes are packaged into secretory vesicles at the “early” TGN, whereas vacuolar cargoes dissociate from VSRs at the “late” TGN due to the low concentration of Ca^2+^ and low pH achieved by the action of TGN-localized H^+^-ATPase. At the “late” TGN, ligand-free VSRs recycle back to the ER via retromer, and vacuolar cargoes travel to the PVC via maturation of the TGN into the PVC and, finally, to the vacuole [95]. Currently, however, there is no experimental evidence for supporting the Ca^2+^-based model in plants. On the contrary, there were several reports supporting the bulk flow secretion. For example, sporamin lacking the ssVSS and sec:GFP, a chimeric construct consisting of the leader sequence of binding protein (BiP) and GFP, were secreted into the apoplast [96,97]. sec:GFP may not have any Ca^2+^-binding property which is required by this model. Thus, the Ca^2+^-based model needs to be further tested in terms of both secretion and vacuolar transport in the future.

## 5. Conclusions and Perspectives

VSR-mediated trafficking of soluble vacuolar proteins through the endomembrane compartments has been studied extensively in plant cells. However, many questions remain unanswered. In fact, there are two different models for VSR-mediated trafficking of soluble vacuolar proteins in plant cells. One key issue is where cargoes are sorted by the receptors, VSRs, and then released from the VSRs. Another key issue is how cargoes are transported from the TGN to the PVC. Currently, it appears that more lines of evidence support Model I than Model II; however, further studies are necessary to exclude either of these two models or to support both of them. More direct evidence for cargo binding to, and release from, VSRs at their respective locations will be important for supporting both models. In addition, for Model I, more direct evidence is necessary for the involvement of clathrin at the TGN. For Model II, little information is currently available about the molecular machinery involved in VSR-mediated trafficking of soluble vacuolar proteins; thus the identification of these molecular factors will provide additional support for this model. Information about the exact pH and Ca^2+^ concentration at the Golgi, TGN, and PVC may also be crucial for explaining experimental results and designing future experiments.

Another intriguing question about the role of VSRs in vacuolar trafficking is how they are involved in trafficking proteins to the lytic vacuole and PSV in both leaf and seed cells. Lytic vacuolar proteins and PSV proteins have the sorting signals ssVSSs and ctVSSs, respectively. Thus, further studies are necessary to elucidate how VSRs function in both pathways with two different types of sorting signals.
